# Ethical and social implications of implementing polygenic embryo screening into clinical care: A scoping review

**DOI:** 10.1016/j.gimo.2026.104405

**Published:** 2026-05-27

**Authors:** Betty Cohn, Dorit Barlevy, Gabriel Lázaro-Muñoz

**Affiliations:** 1 Institute for Public Health Genetics, University of Washington, Seattle, WA; 2 Center for Medical Ethics and Health Policy, Baylor College of Medicine, Houston, TX; 3 Center for Bioethics, Harvard Medical School, Boston, MA; 4 Department of Psychiatry, Massachusetts General Hospital, Boston, MA

**Keywords:** Ethics, Polygenic embryo screening (PES), Preimplantation genetic testing for polygenic conditions/traits (PGT-P), Scoping literature review

## Abstract

**Purpose:**

Polygenic embryo screening (PES), or preimplantation genetic testing for polygenic conditions/traits, is an emerging application of polygenic risk scores within in vitro fertilization. Marketed commercially to prospective parents with limited professional guidelines, PES raises ethical and social concerns. To date, no scoping review has synthesized the ethical landscape of PES. Our study addresses this gap by mapping ethical and sociocultural issues across academic literature.

**Methods:**

Following Preferred Reporting Items for Systematic Reviews and Meta-Analyses guidelines, in September 2024, we searched PubMed, Web of Science, and PhilPapers, yielding a total of 399 articles. After abstract screening and full-text review, the final dataset included 54 articles. Using the 4 principles of biomedical ethics - beneficence, autonomy, non-maleficence, and justice–as an a priori codebook alongside inductive thematic coding, we analyzed how PES is ethically evaluated.

**Results:**

Under beneficence, concerns emerged over clinical validity/utility and uncertain net benefit. Autonomy-related discussions emphasized informed consent and counseling challenges, decision-making shaped by incomplete or biased information, appeals to procreative beneficence, tensions surrounding reproductive autonomy, and respect for the autonomy of future offspring. Non-maleficence concerns included pleiotropy, increased medical risks from in vitro fertilization pursued solely for PES, psychological harms, and clinician-driven embryo selection. Justice subthemes included unequal access to PES, fears of eugenics, stigmatization/discrimination, and potential demographic impacts. Sociocultural perceptions addressed distinctions between conditions and traits, definitions of “healthy,” and disability and feminist critiques.

**Conclusion:**

Although proponents frame PES as advancing reproductive choice and potential health benefits, critics underscore unresolved scientific limitations and significant ethical and sociocultural concerns. The commercial expansion of PES warrants continued study of its validity/utility and standardized counseling.

## Introduction

Advances in reproductive technologies have expanded the scope of embryo screening and selection in assisted reproduction. In addition to preimplantation genetic testing for chromosomal conditions, also known as aneuploidy (PGT-A), or monogenic conditions (PGT-M), a new genetic testing tool called polygenic embryo screening (PES) or PGT for polygenic conditions and traits (PGT-P), allows people undergoing in vitro fertilization (IVF) to screen embryos for the genetic likelihood of complex conditions, such as psychiatric conditions, cancer, and diabetes, and complex traits such as educational attainment, height, and skin tone.

Unlike PGT-A or PGT-M, PES relies on polygenic risk scores (PRS), which are calculated using data from genome-wide association studies (GWAS). GWAS have identified thousands of genomic variants or single nucleotide polymorphisms associated with disease. In clinical settings, PRSs are intended to identify individuals who may be at elevated genetic risk for common complex diseases, such as heart disease, diabetes, or certain cancers, so that early interventions can be implemented for those at high risk.^1^ However, these data possess certain limitations that makes their use problematic in clinical care and, as discussed below, for embryo selection.

PES is currently not offered as a standard of care in fertility clinics. Professional societies have warned that it is not ready for these purposes.^2^, ^3^, ^4^, ^5^, ^6^, ^7^, ^8^, ^9^ However, the technology is commercially available, and private companies market PES directly to prospective patients.^10^ The market continues to expand, with patients seeking non-professional feedback from online advice columns,^11^ signaling a continuation of commercialization.

To date, there have been no scoping reviews on PES. The only comprehensive review available is by Capalbo, et al, which synthesized epidemiological, clinical, and ethical considerations but did not use systematic methods. Our work, therefore, addresses a critical need for a systematic review to map out the ethical and social implications of PES.

In this scoping review, the findings are organized around key ethical principles (ie, beneficence, non-maleficence, autonomy, and justice) and sociocultural considerations. Within each principle, recurring themes highlight the complex debates surrounding PES. This framework allows for a systematic analysis of the literature across diverse stakeholder perspectives, including clinicians, patients, ethicists, and the public.

## Materials and Methods

### Data collection

**Figure 1:**
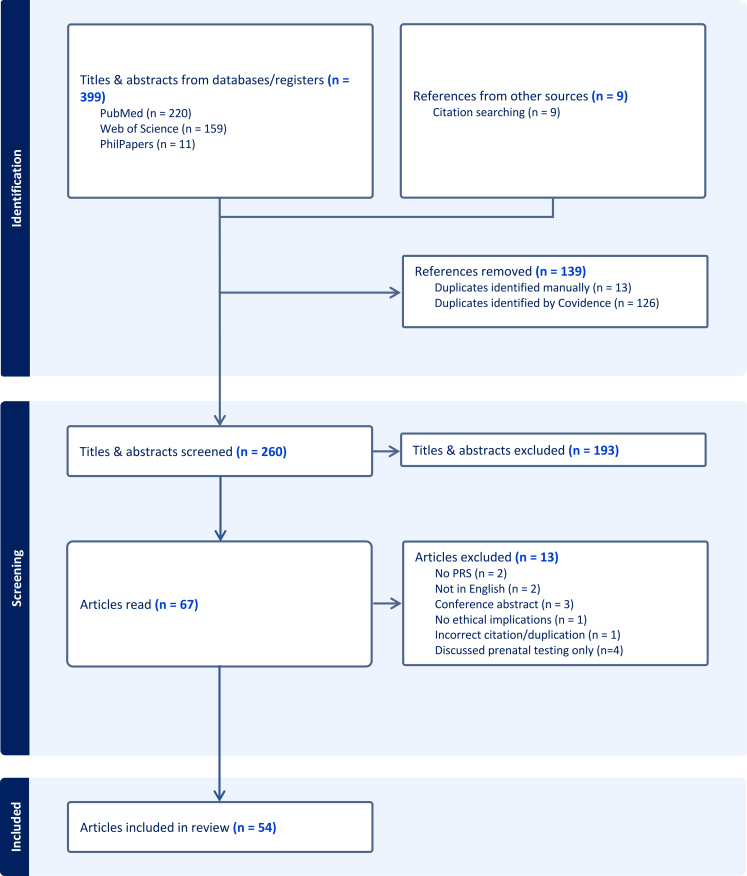
**Flow diagram.***PRS*, polygenic risk score.

We conducted a scoping review based on the Preferred Reporting Items for Systematic Reviews and Meta-Analyses guidelines.^12^ Using the search string “polygenic AND embryo AND ethic,” we searched the online databases of PubMed, Web of Science, and PhilPapers on September 24, 2024, and downloaded all papers. From these searches, we identified a total of 399 academic articles (PubMed [*n* = 220], Web of Science [*n* = 159], PhilPapers [*n* = 11], and 9 additional articles from citation searching). A total of 139 duplicates were removed, leaving 260 unique articles for title and abstract screening. We used Covidence, a software program for systematic reviews, to track our progress throughout each stage of the review. Our exclusion criteria included articles not focused on PES, articles not written in English, conference abstracts, and articles with no mention of ethical implications. Titles and abstracts were screened for relevance, resulting in the exclusion of 193 articles that did not meet the inclusion criteria. The remaining 67 articles were assessed for eligibility through full-text review. During this stage, 13 articles were excluded for not meeting the inclusion criteria, resulting in a final dataset of 54 articles for our analysis. See Figure 1 for a flow diagram that outlines our data collection. This review was not registered, nor was a protocol prepared.

### Data analysis

All documents were coded in Dedoose, a cloud-based qualitative analysis software. One coder (B.C.) coded all documents, and a second coder (D.B.) subsequently reviewed all coded excerpts. Intercoder reliability was calculated as 85%. B.C. and D.B. discussed and reconciled the 15% of coding disagreements.

Coding was conducted using an a priori codebook based on the 4 principles of bioethics: beneficence, non-maleficence, autonomy, and justice.^13^ We used a thematic analysis approach^14^ to identify themes in the documents. This allowed for both deductive coding using the 4 principles of bioethics and inductive coding, which resulted in adding other themes that emerged from the data. Additional codes (sociocultural perceptions) and subcodes were added iteratively until a complete codebook was finalized. See Table 1 for all included papers and the themes and subthemes present in each paper. Article type was categorized as empirical qualitative, empirical quantitative, or other. Other article types included commentary, review, extended essay, viewpoint, insight, special communication, statement, news, essay, special report, news & reviews, open peer commentary, editorial, letter to the editor, comment, perspectives, or opinion.

**Table 1:** Ethical and sociocultural themes and subthemes within academic literature on polygenic embryo screening

Citation	Article Type	Themes																		
		Beneficience		Autonomy					Non-maleficence				Justice				Sociocultural perceptions			
		Subthemes																		
		B1	B2	A1	A2	A3	A4	A5	NM1	NM2	NM3	NM4	J1	J2	J3	J4	SP1	SP2	SP3	SP4
Anomaly et al (2020)	C/R/O		X	X	X	X	X		X								X			
Barlevy et al (2024)	QL		X	X	X	X	X		X	X	X		X	X	X	X	X			X
Brenner & Cohen (2000)	C/R/O	X								X										
Capalbo et al (2024)	C/R/O	X	X	X	X	X	X	X	X	X	X		X	X	X	X	X	X		
Chin et al (2023)	QL	X	X	X	X	X			X	X	X		X			X	X			
Chin et al (2024)	C/R/O	X	X	X			X			X	X		X	X	X	X	X	X		
Denbow & Spira (2023)	C/R/O		X		X		X						X	X				X	X	
Du et al (2023)	C/R/O	X	X	X			X													
Forzano et al (2022)	C/R/O	X	X	X	X		X		X						X					
Fletcher et al (2021)	C/R/O	X	X	X	X				X								X			
Furrer et al (2024)	QN		X				X				X		X	X	X					
Ginod & Dahan (2023)	C/R/O	X	X	X					X	X										
Ginod & Dahan (2024)	C/R/O	X	X	X	X		X			X	X		X		X	X	X			
Grebe et al (2024)	C/R/O	X	X	X	X		X		X	X	X									
Grennell (2020)	C/R/O	X	X																	
Herzig et al (2022)	C/R/O	X												X						
Hyman (2023)	C/R/O		X	X					X				X	X	X					
Johnston & Matthews (2022)	C/R/O		X	X						X			X							
Kakourou et al (2024)	C/R/O	X		X							X		X							
Kamenova & Haidar (2023)	C/R/O	X	X	X	X		X				X			X						
Karavani et al (2019)	QN	X	X	X		X			X				X	X						
Komorowski & Feinberg (2022)	C/R/O		X	X		X	X										X			
Kumar et al (2022)	QN	X	X	X									X							
Kumar et al (2021)	C/R/O	X															X			
Lazaro-Muñoz et al (2021)	C/R/O	X	X	X	X	X	X							X	X		X	X		
Lencz et al (2021)	QN	X	X	X	X		X		X	X				X	X					
Lencz et al (2022)	C/R/O	X	X	X	X	X			X		X		X	X	X			X		
Mahase (2022)	C/R/O	X	X												X					
Makrythanasis et al (2023)	C/R/O	X	X	X	X	X	X													
Martschenko et al (2024)	C/R/O	X	X	X	X		X	X			X		X	X	X		X			
Meyer et al (2023)	QN	X	X	X			X		X				X							
Munday & Savulescu (2021)	C/R/O	X	X	X		X	X		X	X	X		X	X	X	X	X	X		
Neuhausser et al (2023)	QN								X				X				X			
Ogbunugafor & Edge (2022)	C/R/O	X	X	X					X		X		X				X			
Pagnaer et al (2021)	QL	X	X		X	X	X		X	X	X		X	X	X	X	X	X		
Pereira et al (2022)	C/R/O	X	X	X	X	X	X			X			X				X			X
Poli et al (2019)	C/R/O			X							X		X		X					
Polyakov et al (2022)	C/R/O	X	X	X	X	X	X		X	X	X		X	X	X	X				
Raben et al (2022)	QN		X						X				X	X			X			
Rubio & Simón (2021)	C/R/O	X		X																
Rueda & Vallés-Poch (2023)	C/R/O		X	X	X		X			X	X						X			
Siermann et al (2022)	QL	X	X	X	X	X	X				X		X	X	X		X			
Siermann et al (2023)	QL	X	X	X	X		X		X		X		X	X			X			
Siermann et al (2024a)	QL	X		X	X						X			X	X			X		
Siermann et al. (2024b)	C/R/O	X	X	X	X	X	X	X		X	X			X	X		X			
Siermann et al (2024c)	QL	X	X	X	X	X	X		X	X	X	X	X	X	X		X	X		
Tellier et al (2021)	QN	X		X			X		X		X		X			X	X			
Treff et al (2019)	QN	X	X	X			X													
Treff (2020)	C/R/O						X			X										
Treff et al (2020)	C/R/O	X	X				X		X	X			X				X			
Turley et al (2021)	C/R/O	X	X	X			X		X	X	X		X	X	X	X	X			
von Stumm & Plomin (2021)	C/R/O		X							X					X					
Widen et al (2024)	C/R/O	X	X	X																
Zappala et al (2023)	QL	X	X	X		X								X						
Totals		41	44	41	24	17	31	3	24	21	24	1	29	24	22	10	25	9	1	2

*C*, commentary; *O*, Others; *R*, Review.

*Note*: ‘QL’ Empirical research (qualitative article type) and ‘QN’ is Empirical research (quantitative article type). Subthemes: B1 = evidence/evaluation of clinical utility/validity, B2 = uncertain net benefit (based on limited clinical utility/validity and limitations of polygenic embryo screening), A1 = informed consent, counseling, and communication, A2 = decisions based on incomplete or biased information about polygenic risk scores and choice overload, A3 = procreative beneficence, A4 = reproductive (procreative) autonomy, A5 = respect of future offspring's autonomy, NM1 = pleiotropy, NM2 = elective/excessive invitro fertilization for polygenic embryo screening increases the medical risks to patients and offspring, NM3 = psychological harms, NM4 = clinicians deciding for patients, J1 = unequal access to results (exacerbating health disparities and inequalities), J2 = eugenics, J3 = stigmatization/discrimination, J4 = altering population demographics, SP1 = condition vs trait, SP2 = disability, SP3 = feminism, and SP4 = healthy.

## Results

We have outlined subthemes categorized into beneficence, autonomy, non-maleficence, justice, and sociocultural perceptions. For organizational purposes, subthemes were broadly categorized into individual principles; however, they can extend to and involve multiple principles.

### Beneficence

The principle of beneficence refers to acting in others’ best interests. We identified two subthemes under beneficence, which include: (1) evidence or evaluation of clinical utility and clinical validity, and (2) uncertain net benefit (based on limited data for clinical utility/validity and limitations of PES).

#### Evidence or evaluation of clinical utility and clinical validity

Clinical utility refers to the extent to which a medical test improves health outcomes, whereas clinical validity refers to how accurate a medical test is in assessing the likelihood of a future genetic condition.^15^ Papers repeatedly note the need for evidence of clinical utility and clinical validity to demonstrate that PES would be beneficial for parents. Multiple articles argue that there is evidence for clinical utility and clinical validity.^16^, ^17^, ^18^, ^19^, ^20^, ^21^, ^22^, ^23^, ^24^, ^25^, ^26^, ^27^, ^28^, ^29^ For example, Widen et al, who are employees and/or shareholders of a company offering PES, state: “The clinical utility of [PES] has also been clearly demonstrated in several peer-reviewed studies. […] Independent research groups have concluded risk reductions of more than 50% for multiple diseases, including a 72% risk reduction of type 1 diabetes among high-risk families, as well as simultaneous risk reductions of over 40% when using an aggregating polygenic health index.”

However, there is also evidence indicating a current limited or lack of clinical utility because of the availability of retrospective data only (and no prospective data).^5^^,^^8^^,^^19^^,^^25^^,^^26^^,^^30^, ^31^, ^32^, ^33^, ^34^, ^35^, ^36^, ^37^, ^38^, ^39^, ^40^, ^41^, ^42^, ^43^, ^44^, ^45^, ^46^, ^47^ Some authors caution that clinical utility and clinical validity may never be reached because of risk calculations for small sample sizes of embryos from each parental dyad.^4^^,^^5^^,^^18^^,^^20^^,^^25^^,^^44^^,^^48^, ^49^, ^50^, ^51^, ^52^, ^53^ For example, Grebe et al assert that “the clinical utility of PES to reduce disease burden remains ‘unproven’ and must be established through further research and longitudinal studies before the test can be responsibly offered.”

#### Uncertain net benefit (based on limited data for clinical utility/validity and limitations of PES)

PESs uncertain net benefit is often cited as being because of its limitations, including: (1) poor or debated clinical utility and clinical validity,^5^^,^^8^^,^^20^^,^^25^^,^^30^^,^^31^^,^^33^^,^^36^^,^^37^^,^^41^, ^42^, ^43^^,^^46^^,^^52^^,^^54^, ^55^, ^56^, ^57^, ^58^, ^59^ and (2) the current limitations of PRSs (eg, inaccurate scores for embryos of non-European ancestries and the complexity of polygenic traits, with scores not necessarily accounting for environmental and lifestyle factors).^4^^,^^5^^,^^8^^,^^16^, ^17^, ^18^, ^19^^,^^21^, ^22^, ^23^^,^^25^^,^^28^, ^29^, ^30^^,^^32^^,^^37^, ^38^, ^39^, ^40^, ^41^, ^42^, ^43^, ^44^, ^45^, ^46^, ^47^^,^^49^^,^^51^, ^52^, ^53^, ^54^, ^55^, ^56^, ^57^, ^58^^,^^60^, ^61^, ^62^, ^63^ For example, Chin et al recognize these limitations by arguing that “the onset of many polygenic diseases results from the interplay of genetic background with environmental factors. To date, PRSs are derived predominantly from middle-aged or elderly individuals of European ancestry who have developed such late-onset polygenic diseases. Because these individuals have presumably led their lives in a radically different environment compared with future generations of children who are yet to be born, the interactions between genetics and environmental factors that have resulted in polygenic diseases in current study populations will simply not be present for the future generations.”

### Autonomy

The principle of autonomy respects individuals’ right to make decisions that align with their values, free from coercion. Under this theme, we identified 5 subthemes: (1) Informed consent, counseling, and communication; (2) Decisions based on incomplete or biased information about PRS and choice; (3) Procreative beneficence; (4) Reproductive autonomy; and (5) Respect for future offspring’s autonomy.

#### Informed consent, counseling, and communication

Some articles mention a concern about parents’ or individuals’ lack of true informed consent during the decision-making process regarding PES. In part, this is due to healthcare providers’ difficulty in comprehending and their limited time to describe PES accurately and clearly, including its risks and benefits.^5^^,^^8^^,^^16^^,^^17^^,^^20^^,^^25^^,^^30^^,^^31^^,^^44^^,^^51^^,^^54^^,^^61^^,^^62^ Similarly, Johnston and Matthews argued, “it may be unreasonable to assume fertility clinics have the time and resources to help patients fully comprehend the risks and limitations of PES, so that they can give truly informed consent.”

Some authors argue that if parents are fully informed, then they should be able to make the decision that is best for them,^36^^,^^52^^,^^58^^,^^60^^,^^64^ including, forgoing PES completely.^49^ Fletcher et al state that “parents, if they are well-informed, are unlikely to perform [PES]. Assuming that the negative attitudes most Americans have against enhancing traits through gene editing extend to genetic screening, parents are likely to want to screen embryos to avoid disease but are probably against choosing the ‘best’ embryo.”

Various articles highlight the need for (genetic) counseling and a current lack of it for PES. There is concern about the capacity and human resources to do so, given the shortage of (genetic) counselors.^5^^,^^23^^,^^27^^,^^29^^,^^30^^,^^35^^,^^39^^,^^40^^,^^45^^,^^53^ In their qualitative study, Barlevy et al noted that “a few clinicians were unsure how to counsel patients about PES and worried about the lack of data and professional guidance on how to do so.”

During the counseling and communication process, healthcare providers should discuss the limitations of PES (eg, that the chosen genotype for the child may not result in the predicted phenotype), the uncertain utility of PES, and how the information may affect embryo transfer decisions (ie, selection or ranking).^4^^,^^5^^,^^32^^,^^44^^,^^46^^,^^47^^,^^52^ Chin, Lim and Muhsin state that “rigorous and comprehensive counselling by accredited genetic counsellors should be mandated to ensure patients fully understand the technical limitations of PGT-P. Unlike monogenic disorders, there is no clear-cut prevention of transmitting polygenic disease traits, only risk scores that indicate the probability of developing such traits later in life.”

Another article describes additional content that should be discussed during the counseling process, including risk information, lifetime incidence, and how the environment can affect the child’s health outcomes.^5^ The authors argue: “More work is needed to clarify the relation between outcomes that matter to prospective parents and the measure of reduced lifetime incidence of disease presented as a measure of utility in preliminary simulations of benefit. A prospective parent will likely care about the ‘reduced burden of disease over the lifetime’ of the future child, not just whether that individual will get a disease at some time. Even when a more adequate measure of the utility of PGT-P is sufficiently demonstrated so that the service can be legitimately offered, there will still be a host of uncertainties about the actual clinical utility of PGT because of the statistical characteristics of a PRS. This illustrates the complexity of the counseling that will be required for those who are considering using such services.”^5^

Additionally, authors specifically worry that patients do not understand the difference between absolute risk reduction (which can be relatively small) and relative risk reduction (which can be relatively large)^4^^,^^5^^,^^19^^,^^22^^,^^23^^,^^25^^,^^29^^,^^30^^,^^39^^,^^44^^,^^45^^,^^47^^,^^51^^,^^53^ thus negatively affecting their ability to provide true informed consent or make decisions regarding embryo selection. Martschenko et al exemplify this point by arguing that “prospective parents, for instance, may make reproductive decisions using [polygenic score] information (eg, via the growing polygenic embryo selection industry) without understanding the differences between relative and absolute risks. This means that they may mistakenly believe that selecting an embryo with a low-percentile [polygenic score] will dramatically decrease, if not eliminate, their future child’s chances for exhibiting a phenotype, and vice versa.” This is especially because of the fact that companies offering PES emphasize “relative over absolute risk reduction.^24^^,^^25^^,^^30^^,^^42^ For example, “The commercial aspect of PGT-P could lead to presenting PGT-P through rose-colored glasses, eg, by emphasizing relative over absolute risk reduction.”^25^

Several articles argue for reporting both absolute and relative risk, as well as population percentile, numerically and graphically.^5^^,^^21^^,^^29^^,^^30^^,^^57^ Capalbo et al present multiple numeric ways of reporting risk in the following excerpt: “The complexity of PES and the ambiguity in presenting its expected benefits leave room for market pressures. For example, PES could be marketed in either relative (‘50% risk reduction’) or absolute terms (‘0.25%,’ for a disease with a prevalence of 1/200). Unless regulated, companies could choose to emphasize the more impressive relative risk reductions, leaving patients uninformed about the small absolute gains and about their actual risk.”

However, some authors show concern that providers do not understand, do not communicate, or have difficulty communicating the difference between absolute and relative risk to prospective PES users.^5^^,^^18^^,^^19^^,^^23^^,^^30^^,^^37^^,^^44^ For example, Lencz et al state that “communication of risk reduction in relative versus absolute terms can fundamentally alter the perception of physicians and patients regarding the clinical effectiveness of a given procedure. Nuanced communication of the clinical effectiveness of PES could be time-consuming, and sufficient expertise might not be available in standard IVF practice.”

#### Decisions based on incomplete or biased information and “choice overload”

Commonly mentioned barriers to reproductive autonomy and true informed consent with respect to PES are issues around decision making, including incomplete or biased information (eg, lack of knowledge of “lived experiences of people with conditions screened for”)^30^ and “choice overload” (ie, too many factors to take into account when selecting embryos) potentially leading to decisional paralysis and thus not transferring any embryos for implantation.^4^^,^^5^^,^^8^^,^^19^^,^^20^^,^^23^^,^^25^^,^^26^^,^^30^^,^^31^^,^^36^^,^^37^^,^^39^^,^^41^, ^42^, ^43^, ^44^, ^45^^,^^49^^,^^53^, ^54^, ^55^^,^^58^^,^^60^ Some patients describe that while they value being able to make an informed choice, having “too detailed” information is also a barrier to true informed consent. For example, “patients using [PES] could receive risk scores for multiple conditions, which can lead to complicated choices for prospective parents if all embryos are at risk and thus ‘affected’ in a way. The options could lead to ‘information overload’ and a ‘paradox of increased choice.’”^25^

Two articles offer a way to diminish choice overload by providing an “overall ‘health score’” so that parents aren’t assessing risk of multiple diseases at once.^5^^,^^30^ Grebe et al describe “some [PES] tests currently offered include a combined risk score for multiple disorders, weighting each individual PRS to produce a single multi-trait PRS. […] This approach may be helpful to some parents.”

#### Procreative beneficence

Procreative beneficence is the argument that “one should select the child, of the possible children they could have, who is expected to have the best life, or at least as good a life as the others, based on relevant, available information.”^65^ This ethical consideration is often used in support of PES and in practice means that if the parent(s) can choose the embryo that will be transferred for implantation, then they should give a future child the “best possible life.”^8^^,^^17^^,^^23^^,^^30^^,^^37^^,^^40^, ^41^, ^42^, ^43^, ^44^, ^45^^,^^47^^,^^60^^,^^62^ For example, Zappala et al state “for their advocates, PGT-P allows parents to endow their offspring with ‘their best genes,’ which is often presented as a moral imperative.”

For some, the best embryo may be one in which the parent(s) seek to “prevent diseases or avoid unwanted traits” which would also “improve the child’s well-being,” quality of life, and result in a “healthier” child.^20^^,^^31^^,^^41^, ^42^, ^43^^,^^54^ However, the “best” embryo may not lead to a pregnancy,^45^ resulting in a difficult choice for the parent(s).^60^ As Siermann et al describe in their qualitative study, “(PGT-M/structural rearrangements [SR] patients) raised questions regarding what the difference between the ‘best’ and ‘worst’ would be, and how people would feel if the ‘best’ embryo did not lead to pregnancy. Moreover, various participants said that, generally, people’s priority is embryos that are most likely to lead to pregnancy, rather than picking the ‘healthiest embryo.’”

#### Reproductive (procreative) autonomy (making decisions about one’s own reproductive health)

Reproductive autonomy is another subtheme that was invoked often in support of PES use, given the general deference society has towards parents choosing how they want to reproduce.^16^^,^^20^^,^^21^^,^^23^^,^^25^^,^^27^^,^^28^^,^^30^^,^^37^^,^^39^^,^^40^^,^^42^, ^43^, ^44^, ^45^, ^46^^,^^51^, ^52^, ^53^, ^54^, ^55^, ^56^^,^^58^^,^^60^^,^^62^^,^^66^ Munday and Savulescu champion this concept by articulating that “there should be no governmental interference in reproductive decision-making. Prospective parents should be free to adopt or reject the technology as they wish […] this is in line with modern genetics, which focuses on providing information and opportunity, eschewing coercion.”

However, too many or too difficult choices (“choice overload”) or social pressure could undermine one’s reproductive autonomy.^19^^,^^25^^,^^30^^,^^36^^,^^41^ For example, Capalbo et al argue: “Ranking embryos based on their risk profiles for 10 to 20 diseases might be overwhelming to patients. This is especially difficult given that no single embryo is expected to have a consistently low risk for all diseases screened. Choice overload is known to hamper autonomous decision-making rather than enable it. Indeed, a few prospective parents have elected not to transfer any embryo, providing anecdotal evidence of choice-induced paralysis during PES.”

Additionally, there is debate over constraints or limits to one’s reproductive autonomy, specifically whether clinician concerns about patient safety or lack of clinical utility can override an individual’s choice to utilize PES.^4^^,^^5^^,^^30^^,^^54^ Forzano et al state that there are “tensions with other parameters [… including] viability scores and implications for the complex counseling profession, especially when the values of professionals and customers for embryo ranking do not match.”

#### Respect for future offspring’s autonomy

Three articles consider the future child’s potential desire not to know their genetic information. Without considering the future child’s desires, the authors argue that parents are violating the child’s autonomy and privacy.^30^^,^^39^^,^^44^ Siermann et al state, “the rights, privacy, and autonomy of the child have to be considered. The child could arguably have a right to this information about their health risk. It is also possible that the child might not want to know information their health risk, especially about late-onset conditions or conditions without treatment options.”

### Non-maleficence

The principle of non-maleficence focuses on avoiding or preventing harm to others. In our study, we identified the following subthemes: (1) pleiotropy, (2) elective or excessive IVF for PES increases the medical risks to patients and offspring, (3) psychological harm, and (4) clinicians deciding for patients.

#### Pleiotropy

Many articles note the concern over pleiotropy (or, more accurately, antagonistic pleiotropy) because screening and selecting for one phenotype perceived as “positive” might result in increasing risk for a phenotype perceived as “negative.”^-32^,^40^, ^41^, ^42^,^45^,^49^,^51^,^54^,^57^,^60^,^63^,^67^ As Hyman describes, “because of poorly understood pleiotropy, selection against several mental disorders, such as bipolar disorder, obsessive-compulsive disorder, anorexia nervosa, and autism spectrum conditions, may also select against creativity, cognitive abilities, academic attainment, and academic achievement (measured using grades in college). Conversely, selecting for cognitive ability and academic attainment may also select for some of these conditions. What is important is that these traits share alleles; the identity of the shared alleles is currently unknown, and the possibility of getting results opposite to what is desired cannot be judged at present.”

It’s unclear whether healthcare providers consistently describe to patients that the risk of some “undesirable” conditions are correlated with some “desirable” phenotypes before they decide which genotypes to screen for.^4^^,^^5^^,^^21^^,^^28^^,^^30^^,^^32^^,^^42^^,^^63^ Not sharing this relevant information to (prospective) parents negatively impacts their ability to give reasonably informed consent, thereby betraying the principle of respect for patient autonomy.

Nonetheless, many diseases are correlated with each other, meaning that screening to avoid one will also avoid another.^5^^,^^8^^,^^27^^,^^28^^,^^40^, ^41^, ^42^^,^^49^^,^^63^ Grebe et al reiterate this point by noting, “advocates of PGT-P have countered that most diseases that are related genetically are positively correlated, such that selecting against one disease may reduce the risk of many diseases.”

#### Elective or excessive IVF for PES increases the medical risks to patients and offspring

In a survey of the United States’ general population, about a third of respondents said that they would consider IVF for the specific purpose of using PES.^56^ However, several potential medical risks to both patients and offspring are cited as potential harms for fertile populations who electively undergo IVF to use PES.^5^^,^^23^^,^^30^, ^31^, ^32^^,^^41^^,^^42^^,^^44^^,^^48^^^,^^^51^, ^52^, ^53^, ^54^^^,^^^58^^^,^^^59^^^,^^^61^^^,^^^66^

For patients, several risks have been cited, including physical harms from IVF, such as ovarian hyperstimulation,^30^ bleeding,^30^ infection,^30^ pain,^30^ and injury,^30^ as well as pregnancy complications (eg, preeclampsia, hypertensive disorders, higher placental volume, postpartum hemorrhage, atypical placentation, reduced uterine artery pulsatility index, marginal cord insertion accessory lobes, vascular malperfusion, umbilical cord, and subchorionic thrombi) and prolonged discomfort.^19^^,^^23^^,^^30^^,^^53^

For (future) children, several physical risks to the children are possible because of using IVF for the purposes of PES, including: low birth weight,^30^^,^^42^^,^^52^ preterm birth,^19^^,^^30^^,^^32^^,^^42^^,^^52^^,^^53^ respiratory distress syndrome,^32^ pulmonary hypoplasia in preterm infants,^32^ infections,^32^ neonatal hemorrhage,^4^^,^^32^ birth defects (eg, cardiac defects, imprinting disorders),^23^^,^^40^^,^^42^ increased risk of early neonatal mortality,^52^ and increased risk of adult-onset diseases (eg, cancer, diabetes, and cardiovascular disease).^4^,^28^ As Polyakov et al write, “to undertake fertility treatment for the sole purpose of selecting an embryo that has a marginally lower risk of developing a late-onset polygenic condition appears counterproductive and is likely to result in an overall reduced chance of a healthy life. A similar argument can be made in relation to trophectoderm biopsy, which itself appears to produce higher risks of adverse outcomes in offspring. Therefore, an offspring that is born because of PRS-[embryo selection], where assisted reproductive technology is undertaken for the singular purpose of embryo selection based on this technology, may end up having a less healthy life compared with a naturally conceived individual.”

Some argue that clinicians who support this choice are promoting a choice where the risks outweigh the benefits for fertile populations.^5^^,^^31^^,^^42^^,^^45^^,^^53^^,^^54^ Specifically, Chin et al state: “Unnecessarily putting healthy and fertile patients to such grueling medical procedures just for the sake of polygenic testing and selection of embryos through PGT-P must be considered ‘bad medicine’ and clinical malpractice.”

Furthermore, undergoing multiple rounds of IVF ultimately increases the aforementioned medical risks to the patient and offspring, “particularly in poor responders, or if there are no embryos with a low PRS, [and] adding […] increased cost of testing.”^5^ And as Chin et al state, “even for patients with genuine fertility problems, PGT-P would unduly pressure women to unnecessarily undergo multiple IVF cycles to produce excess embryos for polygenic testing and selection. This could add unnecessary stress and confusion to the already difficult and challenging IVF process and encourage patients to discard embryos unnecessarily, thus detracting from the main aim of conceiving a child through IVF treatment.”

#### Psychological harms

Articles also discuss potential psychological harms for parents and children because of PES use, including fears of negative impacts on the parent-child relationship.^5^^,^^22^^,^^25^^,^^27^^,^^39^^,^^40^^,^^42^^,^^44^^,^^45^^,^^53^^,^^54^ For parents, there is the possibility of decisional regret or (moral) distress over embryo selection.^25^^,^^26^^,^^30^^,^^35^^,^^41^^,^^42^^,^^44^^,^^45^^,^^51^^,^^53^^,^^54^^,^^58^ Polyakov et al articulate this point, writing: “The choice made today may not necessarily be the best one in the long run. Parents will have to live with the knowledge that they may have chosen the ‘wrong’ embryo for the rest of their lives. This could potentially result in decision regret and long-term psychological morbidity.” Some articles also note the potential for (prospective) parents’ disappointment in terms of a selected embryo not resulting in a live birth (possibly due to damage to the embryo as a result of the required cell biopsy)^32^,^42^,^52^ or the child not living up to parental expectations.^31^,^36^,^39^, ^40^, ^41^,^45^,^52^,^54^,^56^ Siermann et al maintain: “If PGT-P were to give an idealized image of the health of the child, it could be psychologically more difficult to deal with if the child then developed a certain condition. These possible feelings of blame, expectation, disappointment, and guilt were seen as potentially impacting the parent-child relationship.” For children, there is concern over the potential pressure to live up to parents’ expectations of the chosen PES phenotype(s), or feeling like their parents love them conditionally.^8^^,^^30^^,^^31^^,^^36^^,^^39–41^^,^^43^^,^^45^^,^^52^^,^^56^^,^^64^ Chin, Lim and Muhsin claim: “Children born through PGT- P may feel that their parents do not love them unconditionally as who they are, regardless of their talents and abilities.” Pagnaer et al echo this sentiment, writing: “Opposing views [of PES] feared that children might feel pressured because of high expectations, which could consequently bring a great deal of emotional frustration and harm.”

#### Clinicians deciding for patients

One research study notes Belgian PGT-M/SR patients’ desire to have a healthcare provider make PES embryo selection decisions on their behalf.^45^ As Siermann et al state, “there was strong agreement to leave decision making about selection or prioritization with PGT-P to healthcare professionals (based on their medical knowledge and/or relevant algorithms), to protect prospective parents from difficult choices.”

### Justice

The principle of justice emphasizes the fair distribution of benefits and burdens amid limited resources. We identified 4 subthemes within this category: (1) unequal access to results (exacerbating health disparities and inequalities), (2) eugenics, (3) stigmatization/discrimination, and (4) altering population demographics.

#### Unequal access (exacerbates health disparities and inequalities)

A common justice-related concern is the issue of unequal access to costly PES, which would result in exacerbating health disparities because of the current limitations of PRSs and socioeconomic inequalities.^8^^,^^17^^,^^18^^,^^21^, ^22^, ^23^^,^^25^^,^^27^^,^^28^^,^^30^^,^^31^^,^^35^^,^^39^, ^40^, ^41^, ^42^, ^43^^,^^45^^,^^51^, ^52^, ^53^, ^54^, ^55^, ^56^, ^57^^,^^61^^,^^63^^,^^64^^,^^67^ Martschenko, Matthews and Sabatello clarify this point by stating that “wealthy prospective parents are now able to incorporate [polygenic score] information into embryo selection decisions. Given the disproportionate rates at which [polygenic score] information is accessed by those with privilege—whether financial, racial/ethnic, ableist, educational, or otherwise—it is difficult to imagine how this information will be used to secure rather than stymie justice and equity in clinical and social settings.” One possible solution to this issue is to offer PES as a public service. However, given limited financial resources, the uncertain clinical utility and benefit of PES, and its lack of generalizability to populations of non-European ancestry, this option is practically unlikely in the foreseeable future.^30^^,^^40^

#### Eugenics

Another justice concern that is brought up often is the perpetuation of eugenics—the belief that selecting for certain desirable heritable traits will improve the human population.^68^ In the aforementioned survey of the US general population, over 90% of respondents expressed concern about PES promoting eugenic thinking and practices.^56^ Linked to this concern, is the fear of creating “designer babies,” which also suggests concern about the commercialization of reproduction.^39^, ^40^, ^41^, ^42^, ^43^, ^44^, ^45^^,^^47^^,^^51^^,^^52^^,^^54^, ^55^, ^56^, ^57^^,^^63^ Lázaro-Muñoz et al state: “The application of PES to psychiatric conditions recalls the ugly history of early 20th century eugenics, in which the scientific community designated certain mental conditions (eg, ‘feeble-mindedness’ or ‘imbecility’) as worthy of elimination. While the specter of eugenics has accompanied the development of modern reproductive technologies since the development of IVF and PGT, these concerns are magnified with the advent of PES.” However, Chin, Lim and Muhsin note that this concern is not as prevalent in non-Western countries, where the practices and ideology are still perpetuated or are not perceived in a negative light.

#### Stigmatization/Discrimination

Another concerning potential implication of using PES is stigmatization or discrimination based on certain traits.^4^^,^^8^^,^^19^^,^^26^^,^^30^^,^^38^^,^^40^, ^41^, ^42^, ^43^^,^^45^^,^^51^, ^52^, ^53^, ^54^^,^^56^^,^^64^ For example, Turley et al remark that “another very worrisome use of [PES] would be the selection of traits on the basis of social constructs of race, such as skin pigmentation, hair color, or facial features. Selection based on such traits might reinforce racist conceptions of biological superiority by signaling, either explicitly or implicitly, that certain traits carry value or stigma, possibly amplifying racial prejudice and discrimination.” Additionally, stigmatization or discrimination of conditions screened for during PES could result in further uptake of PES and potential stigmatization or discrimination of those who were born without using PES, as well as those currently living with those conditions.^8^^,^^30^^,^^37^^,^^39^^,^^45^^,^^57^^,^^59^ As Capalbo et al assert, “considerations of stigmatization and discrimination particularly pertain to psychiatric conditions, where recent research has shown that biological explanations have not reduced social exclusion. It was argued that reducing the risk of a disease does not necessarily imply disrespect to people affected by the disease. However, others have argued that, although the intention of screening may not be disrespectful, this does not prevent a stigmatizing effect in society or tangible emotional harm for disabled people.”

#### Altering population demographics

Several articles also note the concern that using PES for embryo selection could potentially lead to alterations in population demographics, including (1) changes to the sex ratio because male embryos have a greater genetic predisposition to polygenic conditions (eg, “because more polygenic disease conditions such as schizophrenia, heart disease, atrial fibrillation, stroke, prostate cancer, and type 2 diabetes are significantly more prevalent in males than females. The wide-scale application of PGT- P may alter the sex ratio, resulting in an unexpected and undesirable demographic consequences, because female embryos are likely to have a more favorable PRS than male embryos”),^31^ (2) decrease in genetic and phenotypic diversity (eg, “by eliminating disabilities and biological diversity, we risk losing differences between people, and thus also sources of creativity”),^41^ and (3) exacerbation of social disparities^27^^,^^30^^,^^31^^,^^40^, ^41^, ^42^^,^^51^, ^52^, ^53^, ^54^ (eg, “some bioethicists have even posited that over several generations of cumulative genetic enhancement, this may lead to permanent stratification of mankind into two classes of genetically advantaged ‘haves’ and ‘have-nots’”).^31^

### Sociocultural perceptions

In addition to the 4 bioethical principles, sociocultural perceptions describe perceptions and evaluations of PES based on social and cultural values. Within this framework, we identified 4 subthemes: (1) condition vs trait, (2) disability, (3) healthy, and (4) feminism.

#### Condition vs trait

Some articles differentiate between the acceptability of PES depending on whether it is done for medical conditions or non-medical traits.^22^^,^^23^^,^^27^^,^^28^^,^^30^^,^^31^^,^^37^^,^^39^, ^40^, ^41^^,^^43^^,^^45^^,^^49^, ^50^, ^51^, ^52^^,^^54^^,^^58^^,^^60^^,^^62^^,^^63^^,^^67^ However, this distinction may not be so clear-cut. As Barlevy et al note in their qualitative study, “some clinicians and patients’ responses, suggested that the distinction between conditions and traits can be blurry. They occasionally mentioned obesity or achondroplasia when discussing screening for physical traits, autism, Down syndrome, or learning disabilities when discussing screening for intelligence.”

Although one article argues that, in the spirit of patient autonomy, parental requests to screen and select embryos for certain desired non-medical traits should be respected,^53^ others argue that parents should not be permitted to do so because these are trivial aspects and indicative of perfectionistic or consumer tendencies.^30^^,^^44^^,^^45^^,^^52^ One such article provides the following description: “The conditions PGT-P could be offered for were seen by several [PGT-M/SR users] as constituting a ‘grey zone’ regarding the seriousness of the conditions. PGT-P was associated with potentially going too far and being a slippery slope to testing for less serious/non-medical indications, which participants were fearful about. Non-medical screening, as well as broad screening for multiple indications, was associated with striving towards perfection, which was seen as being in line with such tendencies in society more widely, such as beauty ideals. Participants related this to creating children ‘à la carte,’ which they viewed as unethical and leading to ‘a twisted society.’”^45^

#### Disability

A few articles discuss disability in terms of parent perceptions of disability and how these affect their decisions to use PES.^40^^,^^52^^,^^55^ Parents who view disability primarily through a medical lens may be more likely to pursue PES to reduce the likelihood of having a child with an increased risk of a polygenic condition, whereas those who adopt a social or identity-based view of disability may reject the use of PES out of concern, that it devalues the lives of people with disabilities. Some articles discuss how PES may affect the disability community in terms of further stigmatization and discrimination.^8^^,^^26^^,^^30^^,^^37^^,^^40^^,^^41^^,^^45^^,^^55^ For instance, Lázaro-Muñoz et al note: “[Screening for and selecting against embryos with conditions] are issues that members of the disability community, including advocates of individuals with Down syndrome, among others, have previously addressed in the context of earlier genetic technologies, including PGT-A and PGT-M.”

#### Healthy

Two articles mention the subjectivity of what defines a “healthy” child.^23^^,^^54^ Barlevy et al describe that “severity and definitions of health loom large in discussions of PES. Previous studies report that condition severity is a main factor in deciding whether to use PGT, and the ability to control or improve the health of one’s future child(ren) is the main motivation for using it. However, the constitution of severity and health is debatable. Though some scholars believe that consensus on defining these terms is impossible, others propose developing an adaptable framework that incorporates biomedical, social, and personal meanings. Either way, the prospect of PES invites clinicians, IVF patients, and all of society to contemplate the meanings of these concepts.”

#### Feminism

One article analyzes the ethical implications of PES from a feminist perspective. Its authors emphasize the burden of responsibility on the (future) pregnant person. This perspective is also applicable to assessments of prenatal genetic testing and IVF in general. Denbow and Spira assert, “polygenic screening centers the individual decisions of the prospective pregnant person as a driving force in the health of future communities, and the future of populations and worlds. In doing so, it expands upon the logic that underpins prenatal testing, which Roberts has argued ‘reinforces biological explanations for social problems and places reproductive responsibility on women, thus privatizing remedies for illness and social inequity.’ This is striking, given that the bodies under consideration are not yet pregnant. It is also striking, given the unprecedentedly expansive range of conditions polygenic screening claims to guard against.”

## Discussion

We analyzed the literature on the ethics of PES using the 4 bioethical principles of beneficence, autonomy, non-maleficence, and justice, as well as sociocultural perceptions of PES and related aspects of its use.

PES is often framed as expanding patients’ reproductive autonomy and aligning with procreative beneficence.^17^^,^^23^^,^^40^ Such framing supports the ethical use of PES. However, patient autonomy can be compromised by choice overload, incomplete or biased information, and limited comprehension of probabilistic risk.^30^^,^^37^^,^^49^ Additionally, the use of overall embryo health scores and embryo rankings may be unduly directive and inconsistent with the tradition of respect for autonomy as practiced in genetic counseling and medical genetics. Informed consent may also be undermined by clinicians’ difficulty in describing PESs probabilistic outcomes, especially in distinguishing between absolute and relative risk.^19^^,^^25^ Furthermore, a shortage of genetic counselors and a lack of standardized counseling protocols limit adequate communication and thus may undercut informed consent.^5^^,^^27^ Though the use of PES is often considered to enhance patient autonomy, it may ultimately diminish patient autonomy because of its complexity and the current lack of guidelines for counseling about it. Additionally, PES may threaten the autonomy of the future child, potentially violating their privacy or imposing unwanted genetic information.^30^^,^^39^

Sometimes, PES use is defended under the principle of beneficence, as proponents of PES point to retrospective studies showing significant risk reductions.^28^^,^^29^ However, critics emphasize the lack of long-term prospective studies that are necessary for validation, as well as Eurocentric bias in PRSs.^20^^,^^30^ Many papers that highlight the principle of beneficence focus on how PES does not meet the requirements to currently benefit parents or the future child. Several professional societies (eg, American College of Medical Genetics and Genomics, European Society of Human Genetics) also state that PES use is premature and potentially unethical.^5^^,^^20^

In terms of non-maleficence, elective or excessive IVF done to try to genetically optimize one’s offspring with PES embryo selection has the double effect of introducing avoidable medical risks for patients and offspring.^23^^,^^32^ Such use of IVF also touches upon issues of justice, whereby clinician time and expertise are misallocated to pursuits considered medically unnecessary, all in the name of respect for patient autonomy. These concerns contrast with the perspective of Dr. Ellen Goldstein, the co-founder of Beverly Hills Fertility, who states: “I’m most excited about helping more patients, beyond those struggling with infertility.”^69^

Subthemes related to justice go beyond individual concerns by focusing on societal implications. For instance, PES may deepen social inequities because of its financial cost, and the lack of utility and validity of PRSs across populations of non-European ancestry.^39^ Additional concerns include altering population demographics (eg, skewed sex ratios) and reinforcing social prejudices.^31^^,^^51^ However, fears about reductions in human diversity are theoretical and rather unrealistic, depending on the scale of PES use.^70^ Furthermore, commercial market pressures add to fears about PES promoting “neo-eugenics” and leading to “designer babies.”^30^^,^^47^ Though some authors believe coercion to be the main issue of eugenics,^30^^,^^40^ others argue it is the belief that some lives are considered better or worthier than others.^71^

Sociocultural perceptions present within the literature of this scoping review include how society views certain concepts, such as “health,” “disability,” and the delineation between acceptable and unacceptable traits to screen (and select) for in embryos. A recurring critique of PES is that its use embodies the expressivist objection:^72^ selecting against embryos with certain conditions conveys a negative social message about the value of people currently living with those conditions.^30^^,^^37^^,^^40^ This critique emphasizes that selection decisions send a societal message that some lives are “less worth living,” thereby reinforcing stigma and discrimination. As with other prenatal and preimplantation tests, some scholars may oppose PES based on the disability critique of these technologies: a single genetic trait is treated as sufficient grounds to determine whether someone exists, neglecting the broader individuality and worth of the person.^73^^,^^74^ These critiques highlight a certain unease around reproductive technologies that permit genetically-based selection.^75^^,^^76^ Disability advocates argue that PES risks narrowing definitions of what counts as a “healthy” or “valuable” life, further marginalizing disabled communities.^30^^,^^37^

It is surprising that only one paper analyzes the ethical implications of PES through a feminist lens, noting that feminist critiques emphasize the disproportionate reproductive burden placed on women.^55^ Feminist bioethics recognizes that “biomedicine and bioethics are fundamentally gendered in ways that affect both how the life sciences are researched and implemented and how this research and practice are ethically analyzed.”^77^ Although applying a feminist approach to reproductive technologies is not novel (eg, previous applications to IVF and preimplantation and prenatal testing),^77^ it remains relevant for PES, which raises similar moral concerns. Feminist bioethicists have emphasized that reproductive technologies disproportionately affect women more than men. Most recently, De Melo-Martin (2023) notes: (1) women bear unequal risks and responsibilities in reproduction; (2) language around reproductive choices often blames women when outcomes are undesirable; and (3) such technologies can reinforce problematic values, including norms about what constitutes a “good” or “bad” mother and oppressive expectations of maternal responsibility.

### Limitations

This scoping review is limited by our search strategy (including database choices) and the terms used to identify relevant literature. Additionally, we were limited to publications written in English. Lastly, we conducted the search at a single point in time, meaning that literature published after September 2024 was not included in this study. It is possible that we may have missed additional relevant literature because of these limitations.

### Implications/Future directions

Results from this scoping review highlight a persistent research gap in the study of PES. To accurately assess PESs clinical validity (how accurately it predicts genetic risk of conditions) and clinical utility (its ability to improve health outcomes), future research must include prospective studies and rely on diverse GWAS datasets that reflect genetic variation across all ancestries. However, this endeavor is challenging because many polygenic conditions do not arise until mid-to-late adulthood. If PES continues to be offered while such research is being done, it will respect patients’ autonomy if they are counseled on its current limitations. However, PES may continue to undermine justice since it is more relevant for people of European ancestry. Furthermore, to uphold patient autonomy, counseling frameworks should ensure transparent communication about the limitations of PES, including pleiotropy (where one gene influences multiple traits), the probabilistic nature of polygenic risk predictions, and potential psychosocial impacts such as anxiety, guilt, or stigma, and possible negative impacts on future offspring and the parent-child relationship. Additionally, collaboration with disability advocates and feminist scholars is essential to prevent the reinforcement of social inequities and discriminatory narratives about women and disabilities. Finally, there may be a growing need for regulatory oversight to counter the influence of commercial interests with ethical safeguards, ensuring that PES is implemented responsibly and in ways that respect patient autonomy, protect patients and offspring from physical and psychological harm (especially if fertile patients undergo IVF for PES embryo selection), and strive for social justice.

### Conclusion

PES raises complex challenges across autonomy, beneficence, non-maleficence, and justice, and presents a diversity of sociocultural perspectives and critiques. Proponents of PES often justify its use through appeals to autonomy, emphasizing reproductive choice, and beneficence, arguing that it can reduce disease risk and promote well-being. However, critics highlight how PES simultaneously threatens all 4 principles: it may undermine autonomy through limited understanding and commercial influence, blur the boundaries of beneficence and non-maleficence given uncertain clinical benefits, and exacerbate social and distributive injustices. The technology also faces feminist and disability critiques. Although PES is currently commercially available, it remains ethically fraught with debated clinical benefits and significant social risks. Careful evidence-building, counseling, and policy oversight are necessary to minimize the risks of PES/PGT-P.

## Data Availability

No new data were generated or analyzed in support of this research.

## Conflict of Interest

The authors declare no conflicts of interest.
